# A New Permanent Scatterer Selection Method Based on Gaussian Mixture Model for Micro-Deformation Monitoring Radar Images

**DOI:** 10.3390/s24061809

**Published:** 2024-03-11

**Authors:** Weixian Tan, Jing Li, Ting Hou, Pingping Huang, Yaolong Qi, Wei Xu, Chunming Li, Yuejuan Chen

**Affiliations:** 1 College of Information Engineering, Inner Mongolia University of Technology, Hohhot 010051, China; wxtan@imut.edu.cn (W.T.); 20211800128@imut.edu.cn (J.L.); hwangpp@imut.edu.cn (P.H.); qiyaolong@imut.edu.cn (Y.Q.); xuwei1983@imut.edu.cn (W.X.); chunming@imut.edu.cn (C.L.); chen_yj@imut.edu.cn (Y.C.); 2 Inner Mongolia Key Laboratory of Radar Technology and Application, Hohhot 010051, China

**Keywords:** micro-deformation monitoring radar, permanent scatterer, GMM, amplitude deviation

## Abstract

The micro-deformation monitoring radar is usually based on Permanent Scatterer (PS) technology to realize deformation inversion. When the region is continuously monitored for a long time, the radar image amplitude and pixel variance will change significantly with time. Therefore, it is difficult to select phase-stable scatterers by conventional amplitude deviation methods, as they can seriously affect the accuracy of deformation inversion. For different regions studied within the same scenario, using a PS selection method based on the same threshold often increases the size of the deformation error. Therefore, this paper proposes a new PS selection method based on the Gaussian Mixture Model (GMM). Firstly, PS candidates (PSCs) are selected based on the pixels’ amplitude information. Then, the amplitude deviation index of each PSC is calculated, and each pixel’s probability values in different Gaussian distributions are acquired through iterations. Subsequently, the cluster types of pixels with larger probability values are designated as low-amplitude deviation pixels. Finally, the coherence coefficient and phase stability of low-amplitude deviation pixels are calculated. By comparing the probability values of each of the pixels in different Gaussian distributions, the cluster type with the larger probability, such as high-coherence pixels and high-phase stability pixels, is selected and designated as the final PS. Our analysis of the measured data revealed that the proposed method not only increased the number of PSs in the group, but also improved the stability of the number of PSs between groups.

## 1. Introduction

The micro-deformation monitoring radar has multiple advantages, including all-day, all-weather, high-precision, real-time, and wide-area measurement [1,2,3]. Due to advancements in science and technology in recent years, this technology has been widely used in deformation monitoring for areas such as mountains and open-pit mines [4,5,6]. The micro-deformation monitoring radar based on differential interference measurement technology can obtain radar images of the same monitoring area. The images are processed to acquire surface deformation [7,8]. Because the scattering characteristics of each pixel maintain high stability and high coherence for a long time, the influence of the atmospheric phase can be reduced by the phase information of pixels to obtain more accurate deformation. Therefore, it is necessary to screen out high-quality pixels to complete the follow-up deformation analysis [9,10,11].

Since the 1990s, many scholars have studied PS selection methods [12,13,14], including the amplitude deviation method proposed by Ferretti et al. [15] and the coherence coefficient method proposed by Berardino et al. [16]. However, they only consider the pixels’ amplitude and phase information. In addition, some unstable or even incorrect PSs in specific scenes were selected with these methods. Recently, some researchers, both domestically and abroad [17,18,19], have combined conventional methods with the relevant pixel information. Some improved multi-threshold PS selection methods based on the conventional methods proposed. For example, Long et al. proposed a method to select PSs based on four parameters: the amplitude deviation, average coherence coefficient, estimated signal-to-noise ratio (SNR), and displacement accuracy index. Some PSs with high signal-to-noise ratios are selected by this method [20]. However, it still requires two thresholds to select PS. Wang et al. introduced a multi-threshold PS selection method for the coherence coefficient, amplitude deviation index, and phase error [21]. This method automatically determines the threshold, but a pre-set initial threshold in subsequent operations was given. Therefore, corresponding fixed thresholds are set by the existing PS selection methods, which cannot achieve real adaptability [22].

During long-term observation, PSs will disappear or rebirth over time because of the time-variation environment and certain uncontrollable factors, such as human activities, as well as the local vibrations caused by the construction and noise of the system, which affect the monitoring results [23]. Deformation information is estimated by using variables of PSs during inversion. If a fixed threshold is used, the number of PSs in each group will significantly fluctuate. Specific deviation in the quantity and quality of the selected PSs will occur, which results in the reduction of deformation accuracy [24,25]. Therefore, it is necessary to develop an adaptive threshold PS selection method for deformation inversion.

Since Ferretti et al.’s introduction of the SqueeSAR algorithm in 2011 [26], the use of time-series InSAR technology in distributed scatterers (DS) has become a research hotspot for deformation monitoring. The technology selects the pixel by using the statistical distribution function with the same statistical distribution and identifies the statistically homogeneous pixels (SHP) within the pixels’ neighborhood. Its research focuses on DS extraction and phase optimization [27]. SHP selection is a prerequisite for the reasonable description of the DS distribution feature. DSs are spatially distributed irregular surface structures found in regions with bare land and sparse vegetation. Many neighboring pixels are found in the same land area and have similar reflection rates and statistics features. In 2015, Jiang et al. proposed a fast statistically homogeneous pixel selection algorithm. In the background of the normal distribution assumption, the confidence interval logic judgment’s average amplitude is used by the algorithm rather than the above-mentioned assumption test, significantly improving the calculation efficiency [14]. In 2020, Jiang et al. evaluated the impact of associated errors on the DS-InSAR time-series analysis method. By eliminating the correlated deviations, the authors enhanced the construction of the coherent matrix, subsequently applying it to optimize the DS interferometric phase [28]. Scatterers are selected by both PS and DS-InSAR technologies, which rely on the ground’s scatterer characteristics for analysis. DS-InSAR selects homogeneous pixels with similar statistical distributions in a certain area, but it does not select stable pixels in the timing monitoring. However, the proposed method can identify stable pixels during long-term observation.

In this paper, a new PS selection method based on the GMM for micro-deformation monitoring radar images is proposed. This method, based on a clustering idea, used multiple pixels’ information to select high-quality pixels that met our requirements. Via comparison with the conventional selection method, our method not only effectively increased the number of PSs, but also improved the stability of the number of PSs to some extent.

The remainder of this paper is organized as follows: In Section 2, the proposed method is introduced in detail. In Section 3, the effectiveness of the proposed method is verified with the experimental. In Section 4, the proposed method is discussed. Finally, some works are summarized and this study’s outcomes are given.

## 2. Methods

A micro-deformation monitoring radar operates with a zero-baseline configuration and acquires two complex radar images referred to as the master and slave images. The interference phase is obtained from two radar images. For each pixel (x,y) on the radar image, the atmospheric phase changes over time in actual monitoring. At the same time, the noise phase changes during radar work. Therefore, the final interference phase can be expressed as follows [29]:

(1) $$\Delta \varphi_{x,y}=\Delta \varphi_{def_{x,y}}+\Delta {\varphi_{atm}}_{x,y}+\Delta {\varphi_{noise}}_{x,y}$$

where $\Delta {\varphi_{atm}}_{x,y}$ represents the difference value in the atmospheric phase during two observation processes, and $\Delta {\varphi_{noise}}_{x,y}$ is the noise phase. The deformation estimation process properly separates the deformation component from the other components, as shown in (2). The plural expression $\phi_{x,y}$ of a single pixel is defined as follows [30]:

(2) $$\phi_{x,y}=A_{x,y}\cdot \exp(-j\Delta \varphi_{x,y})$$

where $A_{x,y}$ is the amplitude value of the pixel and $\phi_{x,y}$ represents the phase value of the pixel.

Ferretti proposed a permanent scatterer technology that selected high-stability pixels as PSs. The amplitude deviation method estimates amplitude stability to replace the phase stability estimation with the mean and standard deviation of radar images [31]. That is:

(3) $$m_{x,y}=\frac{1}{N}\sum_{k=1}^{N}A_{k_{x,y}}(k=1,2,\ldots ,N)$$

(4) $$\sigma_{x,y}=\sqrt{\frac{1}{N}\sum_{k=1}^{N}\left(A_{k_{x,y}}-m_{x,y}\right)}$$

(5) $$D_{x,y}=\frac{\sigma_{x,y}}{m_{x,y}}$$

where $m_{x,y}$ and $\sigma_{x,y}$ indicate the mean and standard deviation of time sequential amplitude, respectively. By setting the suitable threshold $D_{T}$, if $D_{x,y}$ < $D_{T}$, the pixel can be considered as a PS.

From the definition, the pixels with fewer random phase errors are high-coherence points during long-term observation. The amplitude deviation index $D_{x,y}$ only indicates the stability of the pixel’s amplitude information (including the mean and standard deviation), but it does not meet the phase-stability requirements in some special cases. Therefore, if only the amplitude deviation method is used to select high-coherence points, it will inevitably select points with smaller amplitude deviation indexes but larger phase errors. By analyzing radar images, during the long-term observation of different regions in the same scenario, the quantity and phase quality of each group’s PSs significantly differed from one another by a fixed amplitude deviation threshold. When PSs based on this result are used to calculate later deformation inversion, the accuracy is reduced greatly. 

To solve this problem, the method is mainly based on GMM clustering ideas to replace conventional threshold methods. The GMM is a probability model, assuming that all data points are generated from a Gaussian distribution with unknown parameters. By finding clusters in the cluster not clearly defined in the data set, the data are categorized according to the probability distribution. The main advantages of the GMM are as follows:

① The GMM assumes that the input data of each cluster are generated by one or more Gaussian distributions (also known as normal distribution). By using multiple Gaussian distributions to describe the mixed distribution data, the distribution data fit better. The GMM can adapt to various complex data structures, including data comprising multiple subsets, allowing it to more accurately model complex data structures and multi-mode distributions.

② The ability to describe any oval shape is a significant advantage of GMM. By allowing non-spherical shapes, the GMM more accurately represents the real distribution of data in each cluster. In fact, the flexibility reduces the standard deviation of data in each probability distribution, thereby more accurately describing the data structure.

③ The GMM is used to find the optimized parameters by the Expectation Maximization (EM) algorithm. One advantage of the EM algorithm is that it estimates the model parameters when there is a hidden variable, reflected in the probability of each data point corresponding to every other data point. Using the EM algorithm, the parameters of each cluster are dynamically adjusted by the GMM to optimize the distribution of given data.

④ The estimation of the parameter is usually used through maximum likelihood functions in the GMM. The goal of maximizing likelihood function is to find a group of parameters, so that for a given set of parameters, the union probability of data is the largest and selects the best distribution model suitable for a given data set.

### 2.1. PS Selection Process

**Figure 1:**
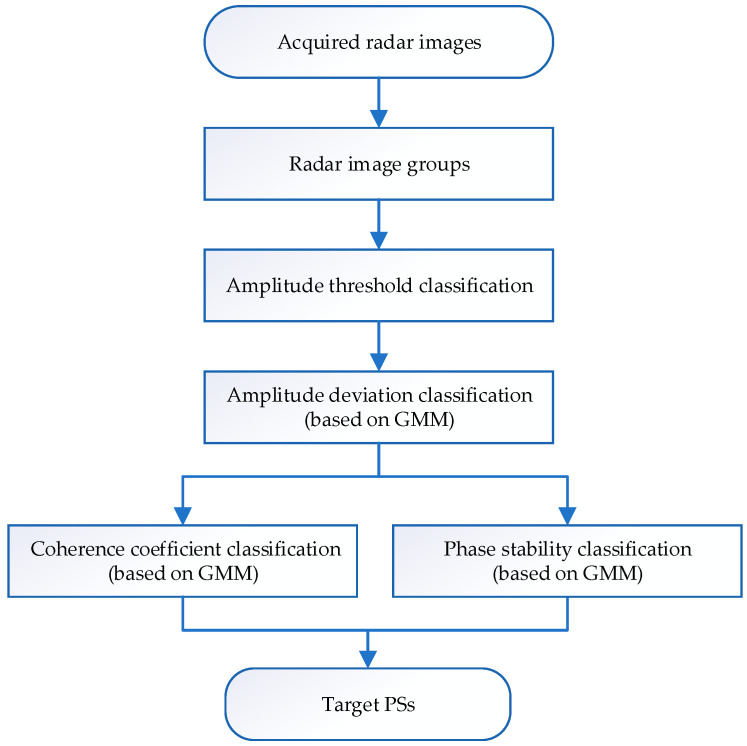
PS selection process flow chart.

The flow chart of the PS selection method proposed in the paper is shown in Figure 1. Firstly, to ensure that the PSs maintain the latest status in the long-term monitoring of the target area, multiple radar images are packed. After group processing, the pixels in each group of images are effectively traversed to improve the timeliness of PSs. Secondly, each pixel’s amplitude information in N radar images and selected PSCs are calculated. Then, the GMM is introduced. Each PSC’s amplitude and phase information are taken as the GMM input data. The data refer to the amplitude deviation index, coherence coefficient, and phase stability of pixels. Using clustering iterations, the GMM parameters are continuously optimized to improve the model’s accuracy and achieve more accurate clustering of PSCs. Finally, higher quality and more PSs are screened.

### 2.2. PS Selection-Specific Process

#### 2.2.1. Radar Image Groups

**Figure 2:**
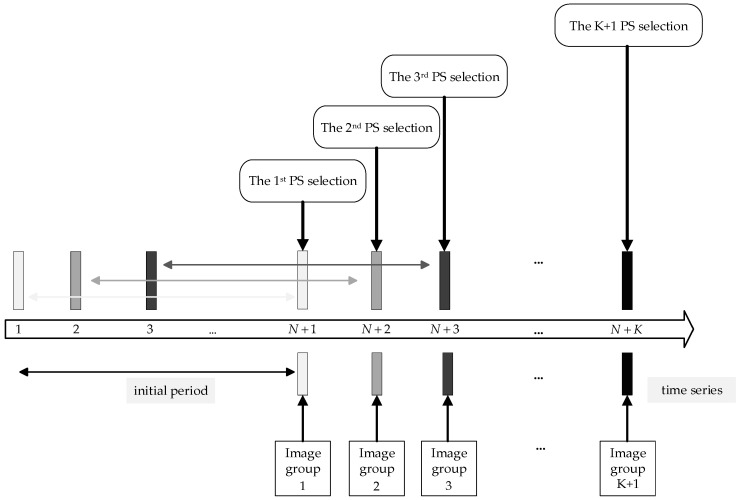
Framework of the radar image groups and PS selection processing.

During long-term monitoring, scatterers’ scattering characteristics continually change over time. Considering that PS selection after collecting all radar images or only some images could be selected in time, PSs are required to ensure the deformation accuracy. Figure 2 shows the framework of the radar image groups and PS selection processing. Each process obtained a certain number of N radar images during PS selection. During the entire monitoring, this strategy ensured that each radar image’s deformation measurement is based on effective PSs in time.

The acquisition time and interval time of the two radar images is relatively short, and the images can be reasonably grouped. Therefore, through group processing, PS selection could be updated in time, improving the real-time of the PSs selection method. This method is significant for the long-term monitoring and timely discovery of possible deformation conditions, improving the deformation accuracy as the deformation information of the observation area is estimated through the deformation of PSs. Therefore, the quantity and quality of PSs are key factors affecting the deformation accuracy. If a large gap exists between the number of PSs in each image, it will seriously affect the deformation accuracy. Thus, the number and quality of PSs need to be comprehensively considered to ensure that the selected PS fully reflects the area’s deformation information and maintains a certain level of consistency between different times.

#### 2.2.2. Amplitude Threshold Classification

For a group of N radar images, the amplitude sequences of each pixel are used as the input data for preliminary screening. Pixels were designated as PSCs. The amplitude information is defined as follows [32]:

(6) $$T=max\{\frac{1}{row\times col}\sum_{x=1}^{row}\sum_{y=1}^{col}A_{k_{x,y}}\}$$

where row and col represent the number of rows and columns of radar images in the complex matrix, and $A_{k_{x,y}}$ represents the amplitude of each pixel in the radar image after radiometric calibration. The minimum value $A_{min}$ of each pixel’s amplitude time series selected from the N group radar images is contrasted with the obtained T. If $A_{min}>T$, the pixel qualified as a PSC to add set A1. Otherwise, the pixel is considered as a non-candidate PS.

#### 2.2.3. PS Optimization

Step S1: Amplitude deviation classification based on GMM

For the set A1, the amplitude deviation index $D_{x,y}$ of the PSCs is calculated according to Equation (5). Then, $D_{x,y}$ can be considered as the input parameter of the GMM clustering. The parameters $\left\{\mu_{i}^{\prime },\alpha_{i}^{\prime },\sum_{i}^{\prime }\right\}$ in Equation (9) are often determined by the EM algorithm for iterative optimization solutions. When the values of three parameters no longer change, the iterations are terminated. Then the optimal parameters’ probability is calculated at the time. Pixels of larger probability values are selected and defined as set A2, and are used as input data for coherence coefficient and phase stability classification.

**Figure 3:**
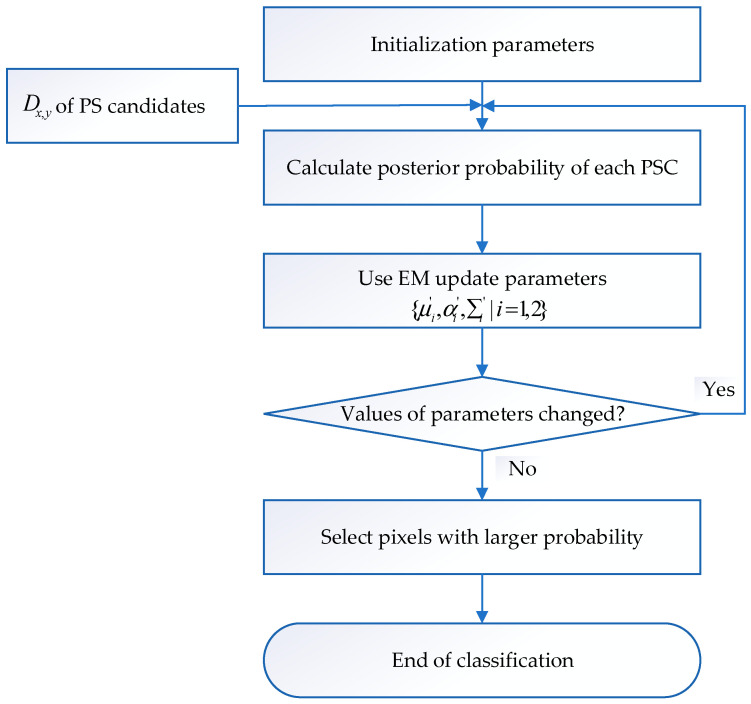
Flow chart of amplitude deviation classification based on GMM.

Specifically, the method makes full use of the amplitude deviation index and coherence information of pixels. The special process of amplitude deviation classification based on the GMM is as follows. The flow chart of amplitude deviation classification based on the GMM is shown in Figure 3.

Step S11: Set two clusters $C_{i}=\{C_{1},C_{2}|i=1,2\}$ and initialize the parameters $\left\{\mu_{i},\alpha_{i},\sum_{i}|i=1,2\right\}$($\overset{2}{\underset{i=1}{\sum }}\alpha_{i}=1$);

Step S12: Calculate the probability distribution of the input data. According to Equation (5), calculate $D_{x,y}$ and subsequently utilize the index as the input data to the probability distributions;

(7) $$p_{i}(D_{x,y}|\mu_{i},\sum_{i})=\frac{1}{{\left(2\pi \right)}^{\frac{1}{2}}|\sum_{i}{|}^{\frac{1}{2}}}e^{-\frac{1}{2}{\left(D_{x,y}-\mu_{i}\right)}^{T}\sum_{i}^{-1}(D_{x,y}-\mu_{i})}$$

Step S13: Calculate the post-probability and utilize $D_{x,y}$ as the input data to obtain the posterior probability $R_{i_{x,y}}$;

(8) $$R_{i_{x,y}}=\frac{\alpha_{i}\cdot p_{i}(D_{x,y}|\mu_{i},\sum_{i})}{\overset{2}{\underset{l=1}{\sum }}\alpha_{l}\cdot p_{i}(D_{x,y}|\mu_{l},\sum_{l})}$$

where μ and ∑ are the mean and covariance matrix, respectively.

Step S14: Take $R_{i_{x,y}}$ as the position of PSCs and $D_{x,y}$ as the input data of EM, update three parameters continuously [33], and obtain new results $\left\{\mu_{i}^{\prime },\alpha_{i}^{\prime },\sum_{i}^{;}|i=1,2\right\}$;

(9) $$\left\{\begin{array}{l} \mu_{i}^{\prime }=\frac{\overset{N}{\underset{v=1}{\sum }}R_{i_{x,y}}D_{x,y}}{\overset{N}{\underset{v=1}{\sum }}R_{i_{x,y}}}\\ \alpha_{i}^{\prime }=\frac{1}{N}\overset{N}{\underset{v=1}{\sum }}R_{i_{x,y}}\\ \sum_{i}^{\prime }=\frac{\overset{N}{\underset{v=1}{\sum }}R_{i_{x,y}}(D_{x,y}-\mu_{i}^{\prime }){\left(D_{x,y}-\mu_{i}^{\prime }\right)}^{T}}{\overset{N}{\underset{v=1}{\sum }}R_{i_{x,y}}} \end{array}\right.$$

Step S15: Execute Step S12–Step S14 in turn and perform GMM cluster iteration. The parameters $\left\{\alpha_{i}^{\prime },\mu_{i}^{\prime },\sum_{i}^{\prime }\right\}$ are updated by each iteration. When three parameter values no longer change, the iterations are terminated, and multiple iterations are ended. Thus, the optimal parameter values of mixed distribution suitable for the data sets are obtained;

Step S16: After the iterations, the probability density is calculated, and the probability is recorded with the optimal parameter values available at the time. Each pixel belonging to the probability of the two corresponding clusters $C_{i}$ is classified. Compared to the two probability values, the pixels in the cluster of larger probability are regarded as low-amplitude deviation index points. According to the above operation, the first PSC is optimized.

The coherence coefficient classification implementation process based on the GMM and phase stability classification based on the GMM are the same as those outlined above. The specific selection is outlined in detail in Step S2.

Step S2: Coherence coefficient and phase stability classification based on GMM

The amplitude deviation method only considers pixels’ amplitude characteristics while disregarding their phase characteristics. This issue may eventually lead to pseudo-PSs emerging, making it inaccurate to only rely on the amplitude deviation method for PS selection.

① For the actual processing, select each pixel according to its phase characteristics [34]. The coherence coefficient γ of each pixel of the radar images is calculated with Equation (10) in each group:

(10) $$\gamma =\frac{\left|\overset{w}{\underset{x=1}{\sum }}\overset{h}{\underset{y=1}{\sum }}M(x,y)S^{*}(x,y)\right|}{\sqrt{\overset{w}{\underset{x=1}{\sum }}\overset{h}{\underset{y=1}{\sum }}{\left|M(x,y)\right|}^{2}\overset{w}{\underset{x=1}{\sum }}\overset{h}{\underset{y=1}{\sum }}{\left|S(x,y)\right|}^{2}}}$$

where M and S are the main and auxiliary image information, w and h are the dimensions of the rectangular sliding window, and ∗ is the complex conjugate multiplication.

As for A2, γ is used as the input data for GMM clustering. Utilize Step S12–Step S14 to execute the iterations and update the parameters. The iterations are terminated until the values of three parameters do not change. Then, calculate the probability of determining the optimal parameters at that time. Pixels with larger probability values are selected as high-coherence pixels, defined as set A3.

② According to (11), calculate the phase stability values $\hat{\omega }$ of these pixels for the radar images in each group [35], that is

(11) $$\hat{\omega }=\frac{\sqrt{(\overset{N-1}{\underset{k}{\sum }}\cos(\varphi_{k}){)}^{2}+\overset{N-1}{\underset{k}{\sum }}\sin(\varphi_{k}){)}^{2}}}{N-1}$$

As shown in A2, $\hat{\omega }$ is used as the input data of GMM clustering. Similarly, Step S12–Step S14 are used for iteration, and three parameters are constantly updated. Pixels with larger probability values are selected as pixels with high phase stability and defined as set A4. The difference between ① and ② is that the GMM clustering input data are different. Thus, take the high-coherent PSs as set A3 and high-phase stability PSs as set A4, and final PSs are selected.

## 3. Experiment and Results

### 3.1. Experimental Area

**Figure 4:**
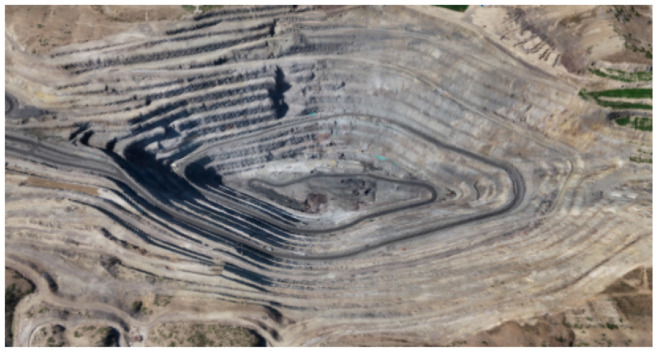
The experimental scene.

This study takes an open-pit mine as its experimental area. The subject of investigation in this study comprises an assemblage of rocks that have accumulated over time, with little to no vegetation coverage. Figure 4 shows the experimental scene. The monitoring system operates in Ku-band with a 500 MHz bandwidth, and its radar range is 100 to 5000 m. The azimuth and range resolutions reach up to 5.4 mrad and 0.3 m, respectively. 

### 3.2. Amplitude Deviation Method

**Figure 5:**
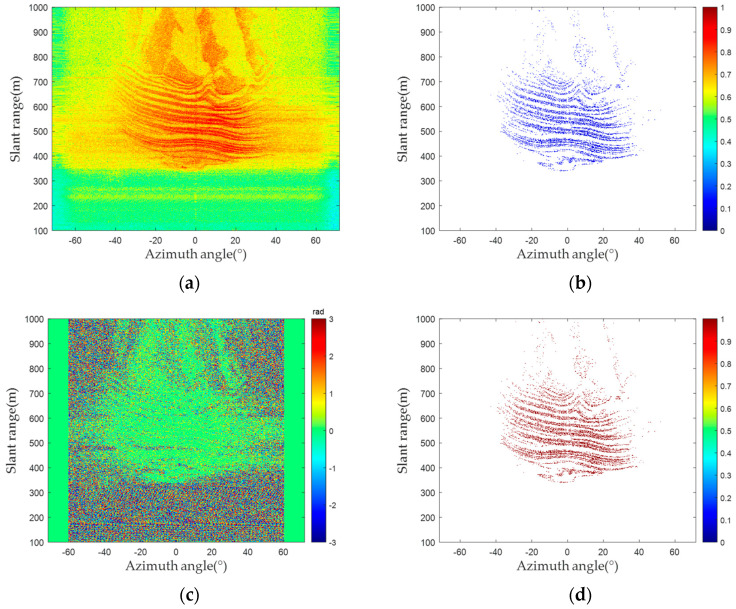
Measured data results: (**a**) radar image; (**b**) amplitude deviation; (**c**) interferogram; (**d**) PS selection result.

A linear scanning micro-deformation monitoring radar technology was used to monitor an open-pit mine. Then, 660 radar images were collected as experimental data. The radar image and amplitude deviation are shown in Figure 5a and Figure 5b, respectively. As shown in Figure 5a, the pixels’ amplitude deviation in the middle area of the image is distributed between 0 and 0.2, indicating that the pixels’ amplitude stability is high. Figure 5c shows an interferogram generated by two radar images with a time baseline of only a few minutes. Due to the short time baseline, deformations do not occur in the scene. As the influence of the atmospheric phase is also small, the interferometric phase is close to 0. However, a more obvious error occurred around the monitoring area, showing that the phase quality of these pixels is low, greatly interfering with the surface deformation accuracy. Thus, selecting high-phase quality pixels is crucial for ensuring the system’s accuracy.

**Figure 6:**
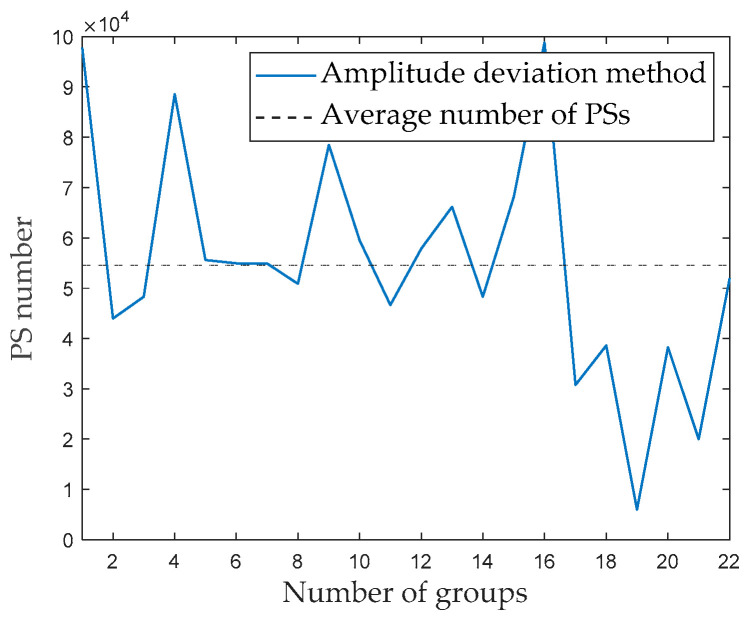
Number of PSs in different groups.

Next, 660 radar images are divided into 22 groups, with 30 radar images per group. The amplitude deviation threshold is set to 0.15. Figure 6 shows the results for the number of PSs in different groups. The abscissa illustrates the number of PSs for different groups; the solid line represents the variation curve obtained by the amplitude deviation method to select the PSs for each group of radar images, while the dashed line represents the average number of PSs for each group. As shown in Figure 6, the number of PSs in each group significantly varied. The number of PSs in the 16th group is the largest, reaching 98,848; the 22nd group had the fewest PSs, reaching only 5962. These two groups of radar images are defined as Group A and Group B.

**Figure 7:**
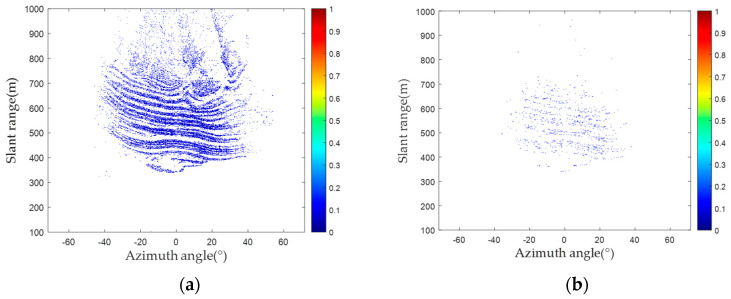
Amplitude deviation of two groups of images: (**a**) amplitude deviation (Group A); (**b**) amplitude deviation (Group B).

**Figure 8:**
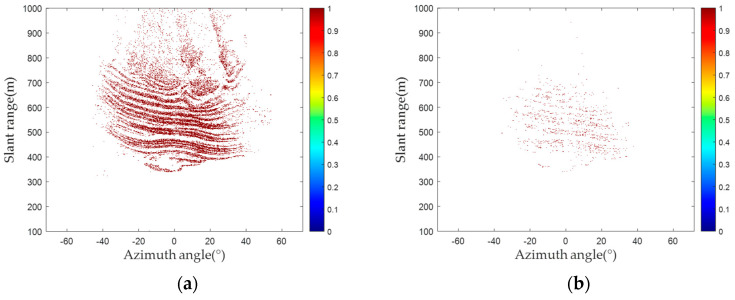

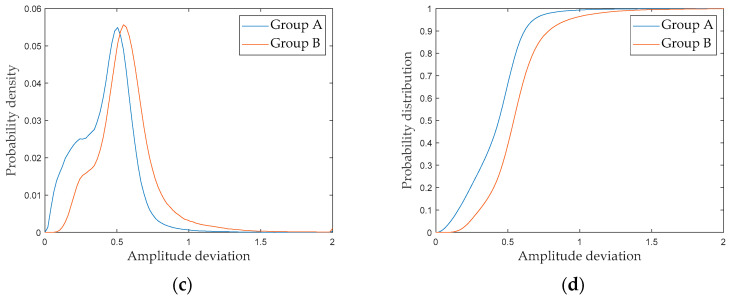
Comparison of amplitude deviation between two groups of images: (**a**) PS selection result (Group A); (**b**) PS selection result (Group B); (**c**) probability density comparison; (**d**) probability distribution comparison.

An analysis of the radar images belonging to groups A and B is conducted. The amplitude deviation for the two groups of radar images is shown in Figure 7. By comparing the amplitude deviation, the amplitude deviation significantly differs between the two groups of images. Figure 8a,b show the results of PS selection for the two groups of images, and Figure 8c,d show the probability density and probability density distribution curves corresponding to the amplitude deviation values of these two groups. Considering the probability statistics, the amplitude deviation index of the images in Group B is greater than that of the images in Group A in Figure 8c. Within Group B, the proportion of the amplitude deviation index exceeding 1.5 is 0.52%, while in Group A, it is close to 0.

Therefore, based on the analysis of the above-mentioned experimental results, PSs are always selected by setting a fixed amplitude deviation threshold. This inevitably leads to significant variations between the PSs groups acquired at different time intervals. Consequently, the deformation results are seriously affected.

### 3.3. PS Selection Method Based on GMM

In accordance with the PS selection method mentioned earlier, a specific group of radar images is analyzed. Firstly, 129,732 points are selected as PSCs in the preliminary screening by the amplitude information method. Next, PSCs are used for secondary classification and serve as input data for GMM clustering. After multiple iterations, larger probability pixels are selected as the input data of the coherence coefficient and phase stability, and 118,876 points are selected according to the standard. Then, the selected pixels’ coherence coefficient and phase stability are used as input data for GMM clustering for further classification. After multiple iterations, pixels with larger probabilities are selected. The high-quality pixels are 66,354 points and 87,378 points, respectively. Finally, 97,721 points regarded as PSs are obtained by taking the union, accounting for about 7.15% of total pixels.

**Figure 9:**
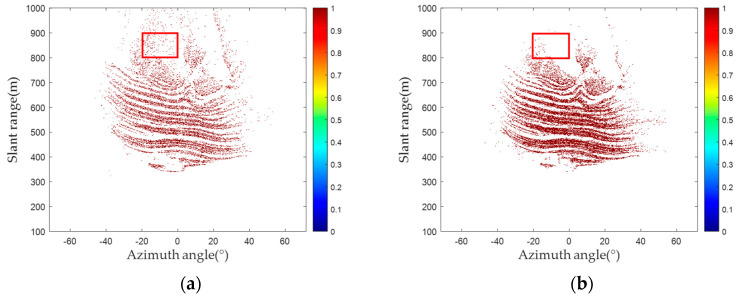

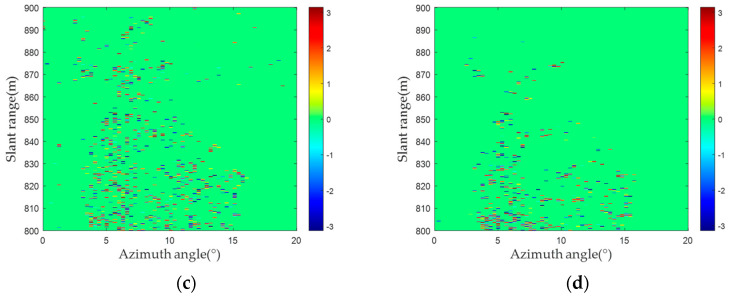
Number of PSs and phase diagrams of PSs in the red frame: (**a**) the number of PSs detected via the amplitude deviation method; (**b**) the number of PSs detected via the proposed method; (**c**) the phase diagrams of PSs in the red frame detected via the amplitude deviation method; (**d**) the phase diagrams of PSs in the red frame detected via the proposed method.

For the ground-based differential interferometry measurement technology, the quantity and quality of PSs play an important role in obtaining accurate deformation. Thus, by contrasting the amplitude deviation and proposed methods, this study focused on the quantity and quality of PSs. Figure 9 shows the number of PSs and phase diagrams of PSs in the red frame detected by the amplitude deviation and proposed methods. As shown in Figure 9, 66,150 PSs are selected by the amplitude deviation method, while 97,721 PSs are selected by the proposed method. As the area had not experienced deformation, the phase of the pixels is considered theoretically stable. Therefore, PSs are evenly distributed and more representative of the phase feature information of the area, and these points located in the phase jump area are no longer considered to be PSs.

**Figure 10:**
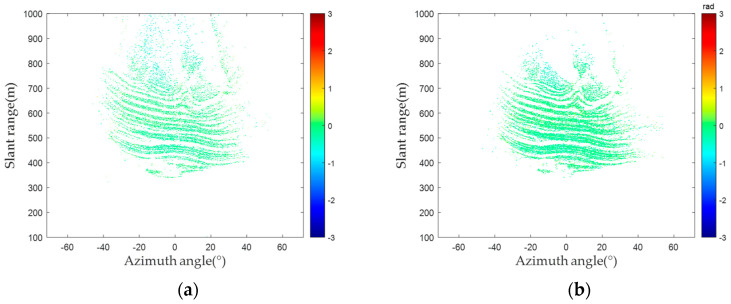
PS selected interferogram: (**a**) amplitude deviation method; (**b**) proposed method.

In each group of 30 radar images, an interference process is applied to two consecutive radar images. Taking the first image as the main image and others as auxiliary images, 29 interferograms (IM ^(1)^ to IM ^(29)^) are generated. As the experimental area mainly consisted of solid rock, no deformations occurred within the period. Theoretically, the interferometric phases of PSs are 0. Figure 10 shows the interferograms acquired through two PS selection methods. The PSs selected by the amplitude deviation method illustrate many pixels with substantial phase errors, as shown in Figure 10. In contrast, the interferometric phases of the PSs selected by the proposed method tend to be significantly closer to 0. Consequently, the proposed method selected PSs that exhibit greater phase-stability characteristics, ensuring the precision of deformation inversion.

**Figure 11:**
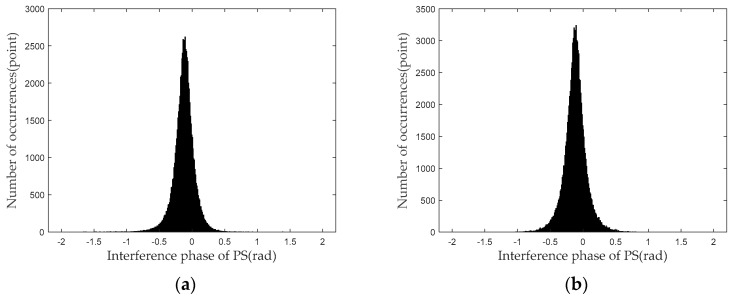
Interference phase distribution histogram of PSs: (**a**) amplitude deviation method; (**b**) proposed method.

Figure 11 shows histograms illustrating the distribution of the interferometric phase for the PSs selected in the group of images by the amplitude deviation and proposed method. As shown in Figure 11, the interferometric phase of each group with time, the phases undergoing gradual change, and some PSs shift to 0. Theoretical analysis indicates that the phenomenon is mainly influenced by the atmospheric phase. The conventional atmospheric phase model is used to effectively compensate for the phase errors caused by the atmospheric phase [36,37,38,39].

The quality of the PSs in the group of images is analyzed. Based on the above theoretical analysis, this study utilizes phase standard deviation after atmospheric phase compensation to quantitatively evaluate the phase quality of PSs. The phase standard deviation is used to quantify the phase deviation distribution of the PSs. The smaller the values, the more stable the selected PS phase [40,41].

**Figure 12:**
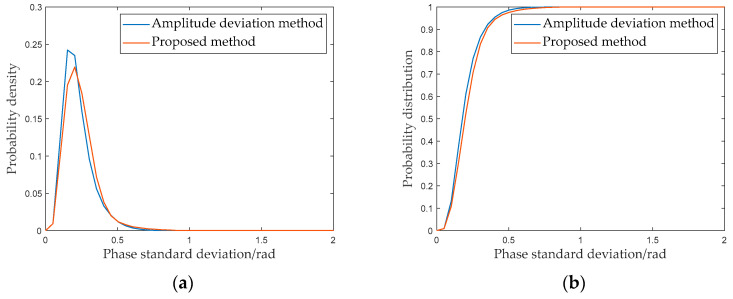
Phase standard deviation of PSs: (**a**) amplitude deviation method; (**b**) proposed method.

Next, the phase standard deviations of the PSs selected with the two methods are shown and analyzed, respectively. Figure 11 shows the distribution of the phase standard deviation at different intervals. Figure 12a is the probability density curve of the phase standard deviations of PSs, whereas Figure 12b is the corresponding probability distribution curve. As shown in Figure 12, a certain difference is present in the probability density curves for the PS groups selected by the amplitude deviation method and proposed method. This phenomenon indicates that the proportions of PSs at different standard deviation intervals are different. Table 1 shows the statistics results for the phase standard deviations of the two PS groups. As shown in Table 1, when the standard deviations were less than 0.5 rad, the number of PSs increased by 46.19%.

**Figure 13:**
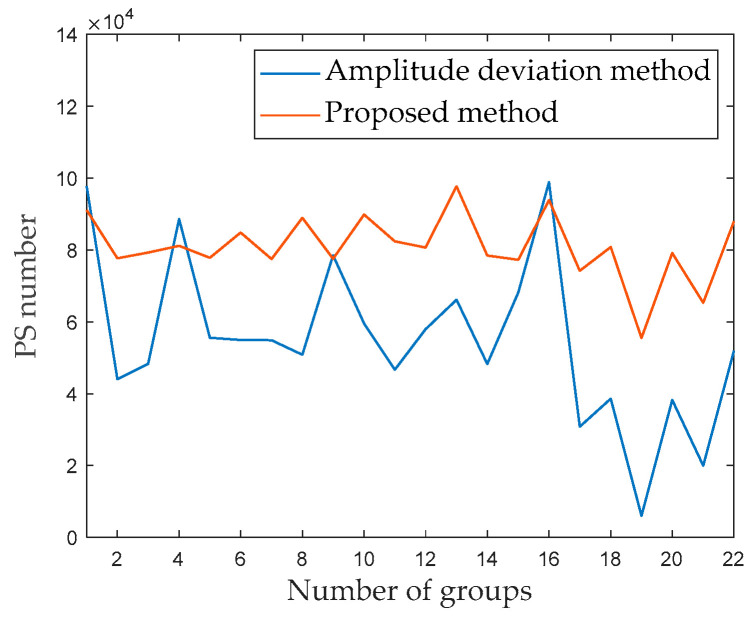
Number of PSs in different groups, as determined using the amplitude deviation method (blue color) and proposed method (orange color).

PSs are selected by the proposed method through processing 22 groups of radar images. Figure 13 shows the number of PSs in each different group. As shown in Figure 13, the number of PSs in each group for the blue curve wildly fluctuates, although the number of PSs in each group for the orange curve fluctuates more mildly. Compared to the experimental results, the proposed method can increase the number of PSs in the group and improve the stability of the number of PSs between each group. Therefore, the proposed method’s effectiveness laid a good foundation for high-precision deformation inversion.

## 4. Discussion

Based on the monitoring data of the ground-based radar, this study processes long-term time-series radar images through group processing, fully considering the pixels’ amplitude and phase information. This paper proposes a PS selection method based on the GMM. Several points are worth exploring:

(1) During the long-term observation of the micro-deformation monitoring radar, the most conventional methods are calculated using the respective indicators through formulas. Then, an appropriate threshold is given to compare with the calculated values to select PSs. However, to deal with different regions in the same scenario, the threshold is repeatedly set by the conventional amplitude deviation method. This operation is tedious in practice and lacks adaptability. Therefore, this study introduced the GMM and proposed a PS selection method to replace the setting threshold by automatic clustering.

(2) The pixels’ amplitude deviation index, coherence coefficient, and phase stability are comprehensively considered and combined with GMM clustering in this study. Depending on the varying characteristics of the PSs required for iterative classification, the selected high-quality pixels’ quantity and quality are accurate, ensuring the adaptability for PSs classification between radar images.

(3) Due to long-term monitoring, the scattering characteristics change over time. Each radar image is utilized for the effective PSs to ensure the deformation results’ validity. Therefore, this study used the form of a group for multiple radar images to constantly update the selected PSs between different groups. The timely update of the PSs improved the real-time PS selection method, ensuring the high-precision inversion of an open-pit mine.

At the same time, it is worth reflecting on some of this study’s disadvantages. The GMM model is very sensitive to the initial value. It may converge to the optimal part, and the convergence speed is slow, which may affect the eventual result. If the optimal initial value at the beginning of the experiment is given, the K-Means (which is repeated and obtained the optimal value) may first be used to identify a rough result. Then, it can be presented as the initial value of the GMM and for subsequent iterations. In addition, it is necessary to find more accurate selection results by training the model to rely on the existence of a large quantity of data.

## 5. Conclusions

Regarding the differential interferometry measurement technology used in the micro-deformation monitoring radar, this paper studies the selection of PSs in deformation monitoring. To address the existing methods’ limitations, a new PS selection method based on the GMM for micro-deformation monitoring radar images is proposed. When conducting the long-term monitoring of an open-pit mine, PSs could be selected by both the amplitude deviation method and the proposed method. A comparative analysis of the quantity and quality of the PSs is performed to validate the proposed method’s feasibility. The experimental data prove that the number of PSs between different groups significantly varied when a fixed amplitude deviation threshold was used. In contrast, the proposed method can effectively increase the number of PSs within each group and improve the stability of the number of PSs between different groups. Notably, the proposed method is adaptable to different regions in the same scenario and does not require us to manually set thresholds.

## Figures and Tables

**Table 1:** Statistics of the phase standard deviation of PSs.

Standard Deviation/rad	Amplitude Deviation Method	Proposed Method	Increased Percentage
	Number	Number	
<0.1	8817	10,661	20.91%
<0.3	57,272	81,587	42.45%
<0.5	65,276	95,424	46.19%

## Data Availability

Data are contained within the article.
